# Primary Biliary Cholangitis Unmasked in Metabolic Dysfunction‐Associated Steatohepatitis: When a Correct Diagnosis Is Incomplete

**DOI:** 10.1002/kjm2.70310

**Published:** 2026-11-02

**Authors:** Yucheng Cheng, Yu‐Hsin Chuang, Ming‐Jong Bair, Jee‐Fu Huang

**Affiliations:** ^1^ Department of General Medicine MacKay Memorial Hospital Taipei Taiwan; ^2^ Department of Internal Medicine, Division of Gastroenterology Taitung MacKay Memorial Hospital Taitung Taiwan; ^3^ Hepatobiliary Division, Department of Internal Medicine Kaohsiung Medical University Hospital, Kaohsiung Medical University Kaohsiung Taiwan

Steatotic liver disease (SLD) coexists in approximately one‐quarter of patients with primary biliary cholangitis (PBC) [1]. Because PBC is frequently asymptomatic and its biochemical abnormalities may overlap with metabolic dysfunction‐associated steatotic liver disease (MASLD), a metabolic phenotype does not exclude coexisting cholangiopathy [2]. We report PBC recognized after nearly 18 years in a patient with metabolic dysfunction‐associated steatohepatitis (MASH).

A 62‐year‐old woman with obesity, Type 2 diabetes, and dyslipidemia had been followed for non‐B, non‐C chronic hepatitis since 2007. Fluctuating aminotransferase elevations and metabolic abnormalities supported MASH [3, 4]; pruritus, jaundice, and sicca symptoms were absent throughout follow‐up. However, γ‐glutamyl transferase (GGT) progressively increased from 85 U/L in 2022 to 166 U/L (4.4× the upper limit of normal) in 2026, while alkaline phosphatase remained normal. Ultrasonography showed no biliary dilatation or extrahepatic obstruction (Figure 1A).

**FIGURE 1:**
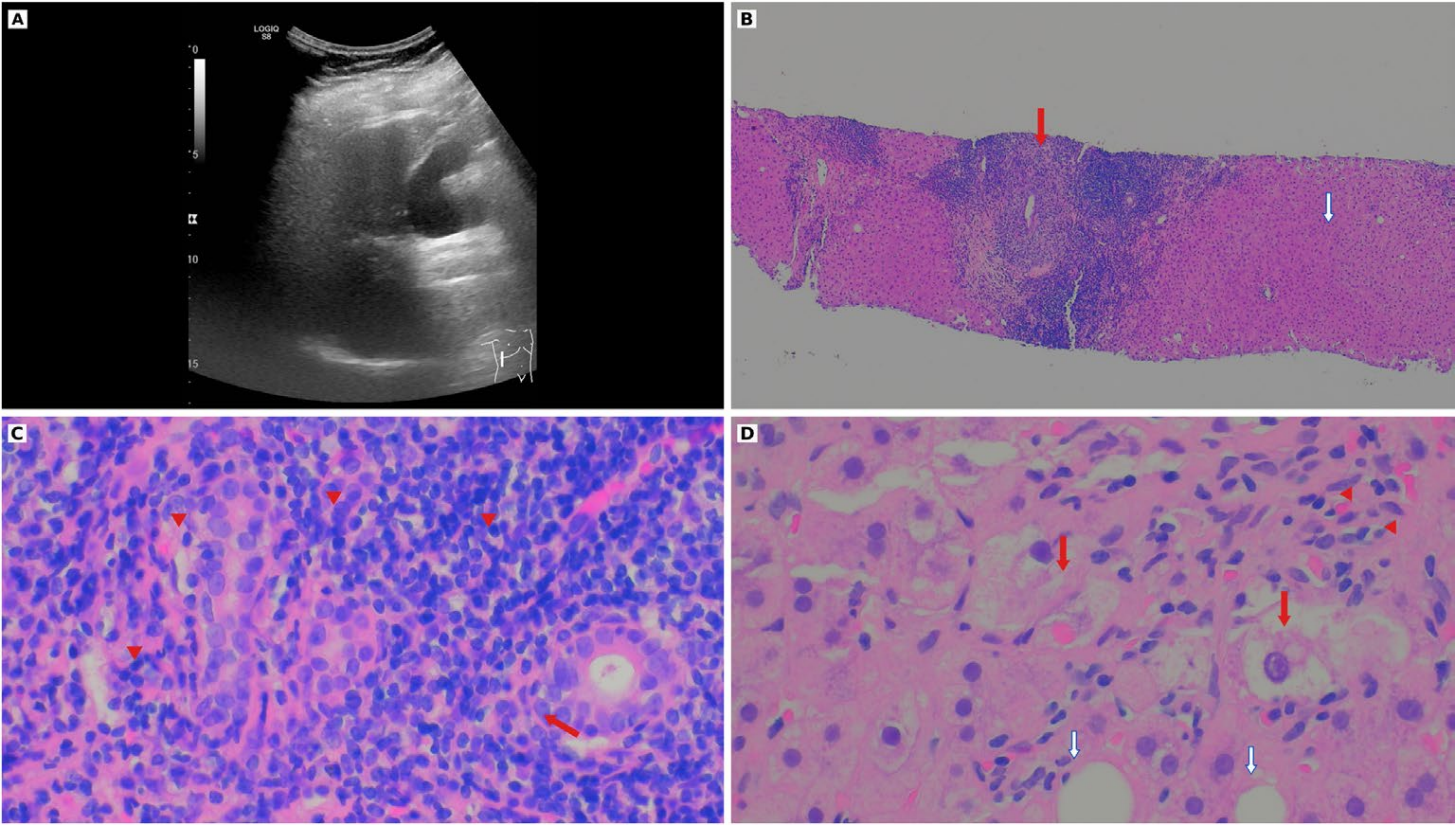
(A) Abdominal ultrasonography revealed coarse, bright hepatic parenchyma consistent with steatotic chronic parenchymal liver disease, without biliary dilatation. (B) Low‐power view (×40) showing a focal florid duct lesion within a markedly expanded portal tract (solid arrow) with surrounding dense portal inflammation. Macrovesicular steatosis is present in the lobular parenchyma (hollow arrow), indicating the coexisting MASH component. (C) High‐power view (×400) demonstrating lymphocytic bile duct destruction (solid arrow) with marked portal inflammation. A dense lymphocytic infiltrate dominates the portal tract (arrowheads). (D) High‐power view (×400) showing the MASH component. Hepatocytes exhibit ballooning degeneration (solid arrows). Macrovesicular fat vesicles are present (hollow arrows). A focus of lobular inflammation is noted (arrowheads). (B–D) Hematoxylin and eosin stain. MASH, metabolic dysfunction‐associated steatohepatitis.

During clinical‐trial screening in July 2026, central review of a liver biopsy (45 mm; 50 portal tracts) confirmed MASH (NAFLD activity score 5; Figure 1D) with advanced fibrosis (F3–F4), but also revealed granulomatous bile duct injury, lymphocytic bile duct destruction (Figure 1C), and a focal florid duct lesion (Figure 1B), collectively identified by three pathologists. Subsequent testing demonstrated antimitochondrial antibody positivity (1:160) and antinuclear antibody positivity (1:640; AC‐7/anti‐sp100‐associated pattern). Despite normal alkaline phosphatase, PBC‐specific serology and characteristic bile duct injury supported PBC [2], establishing coexisting PBC and MASH.

This case illustrates the limitations of metabolic phenotype and routine biochemistry in distinguishing metabolic from immune‐mediated liver injury. Although the GGT elevation was initially attributed to steatotic liver disease, its progressive, disproportionate increase suggested an additional disease process, particularly because GGT may precede alkaline phosphatase elevation in PBC [2]. Histology may reveal superimposed injury patterns: MASH is predominantly lobular, whereas PBC is portal and bile‐duct‐centered [2]. Florid duct lesions are highly specific when present, and concomitant MASH does not obscure characteristic biliary features [2]. PBC‐specific histologic staging systems, such as the Nakanuma system, incorporate bile duct loss and chronic cholestasis in addition to fibrosis [2].

The prognostic impact of concomitant MASLD on PBC remains uncertain: poorer biochemical response to ursodeoxycholic acid (UDCA) and liver‐related outcomes have been reported [1], whereas another study found no adverse effect of nonalcoholic fatty liver disease (NAFLD) on PBC activity, severity, or progression [1]. Nevertheless, recognizing PBC remains clinically important because it establishes an indication for disease‐modifying therapy with UDCA [2].

This case highlights the diagnostic challenge of recognizing coexisting MASH and PBC despite a well‐supported metabolic diagnosis. Persistent or disproportionate cholestatic abnormalities should prompt consideration of coexisting cholangiopathy, including autoimmune serology and histologic reassessment [5].

## Conflicts of Interest

The authors declare no conflicts of interest.

## Data Availability

The data that support the findings of this study are available on request from the corresponding author. The data are not publicly available due to privacy or ethical restrictions.
